# PROTOCOL: The effectiveness of psychosocial interventions for reducing problematic substance use, improving mental health, and improving housing stability for adults experiencing homelessness: A systematic review and meta‐analysis

**DOI:** 10.1002/cl2.1290

**Published:** 2022-11-25

**Authors:** Chris O'Leary, Ligia Teixeira, Esther Coren, Zsolt Kiss, Anton Roberts, Harry Amitage

**Affiliations:** ^1^ Policy Evaluation and Research Unit Manchester Metropolitan University Manchester UK; ^2^ Centre for Homelessness Impact London UK; ^3^ Canterbury Christchurch University Canterbury UK; ^4^ ZK Analytics Oxford UK

## Abstract

This systematic review is part of a broader evidence synthesis which aims to produce two systematic reviews to address a significant gap in the evidence base identified by Luchenski et al. (2018) and by (White, 2018). The first review (which is the subject of this protocol) will use meta‐analysis to examine the effectiveness of different psychosocial interventions in (1) reducing problematic substance use; (2) improving mental health; and (3) improving housing stability for adults experiencing homelessness. The second review (which is covered by a separate title registration and protocol) will be of the experiences of adults experiencing homelessness when accessing or using psychosocial interventions, and will be a qualitative evidence synthesis using thematic synthesis (Thomas & Harden, 2008).

## 1 BACKGROUND

### 1.1 The problem, condition or issue

#### 1.1.1 The significant and increasing scale of homelessness

Homelessness is a major social and public health concern (MacKnee & Mervyn, 2002; Wright, 2017). In recent years, rates of homelessness are reported to have increased in many western countries, although differences in definitions and measures mean that it is challenging to get an accurate overall picture (OECD, 2020). For example, in the United States, the recent *State of Homelessness in America* report stated that in January 2020 over 580,000 were experiencing homelessness, and that rates of homelessness had grown by 2% over the previous year (National Alliance to End Homelessness, 2021). In Canada, around 35,000 people are homeless each night, with between 250,000 and 300,000 experiencing homelessness a year (Gaetz et al., 2016; Wong et al., 2020). Homelessness continues to rise in most EU countries (FEANTSA, 2017). In England, all forms of homelessness rose between 2008 and 2017 (O'Leary & Simcock, 2020), and it is estimated that 280,000 people are homeless in England (Shelter, 2021).

Recent published data suggests that the number of people experiencing street homelessness and who are sleeping rough (unsheltered) in England fell between 2017 and 2021 (snapshot count taken on a single night in Autumn), with a significant fall recorded in 2020. The large drop in 2020 is probably accounted for by government responses to the Covid 19 (DLUHC, 2022), though the reasons for reductions in 2017, 2018 and 2019 are not yet known. In the UK, the proportion of people experiencing homelessness who are sleeping rough is relatively small compared to other forms of homelessness. The upward trend has continued in these other types of homelessness, with the number of households assessed as being statutory homeless (i.e., meet the legal definition of homelessness to whom local housing authorities owe a duty to support) has continued to increase (DLUHC, 2022).

We recognise that homelessness is a complex and multifaceted concept, with differences in how homelessness is understood and experienced, and how these differences are conceptualised and described. During the scoping work for this protocol, a workshop was held with five people with lived experience to consider the research objectives and definitions used. This workshop developed a new definition of homelessness, building on the previous work of Keenan et al. (2020). This definition is set out in the Methods section of this protocol.

There are also ongoing policy and practice debate around the causes of homelessness, and around interventions aimed at preventing and reducing homelessness. In terms of the causes of homelessness, Glen Bramley and Suzanne Fitzpatrick state that there is significant debate between a focus on individual‐level risks or causes, and structural or systemic causes (such as labour market conditions, housing supply, and poverty). These foci vary between countries and over time, though increasingly it is recognised that both might have explanatory power (Bramley & Fitzpatrick, 2018). These debates often influence policy debates around the types of interventions that might address homelessness, and whether these should be focused on structural interventions such as increasing housing supply or reducing poverty, or preventing/addressing homelessness at the level of the individual. Whilst individual experiences are highly likely impacted by the structural contexts in which they arise, this review is focused on individual level interventions.

Homelessness is a traumatic experience, which can have a devastating effect on those experiencing it. Several studies, some of which are cited below, have highlighted that more visible and extreme forms of homelessness are often associated with adverse childhood events (Koh & Montgomery, 2021), extreme social disadvantage (Mabhala et al., 2017), physical, emotional and sexual abuse (Green et al., 2012; Henny et al., 2007), neglect (Mar et al., 2014), low self‐esteem (Seale et al., 2016), poor physical and mental health (Vallesi et al., 2021), and much lower life expectancy compared to the general population (ONS, 2019). People experiencing these more extreme and visible forms of homelessness often experience severe and multiple disadvantages (Bramley et al., 2020) a term which ‘encompasses people who experience some combination of homelessness, substance misuse, mental health problems, and offending, which coincides with many uses of terms such as “multiple needs,” “complex needs,” or “chronic exclusion”’ (p. 391), and need significant levels of professional and service support (Dobson, 2019). They are increasingly the focus of policy interest, both here in the UK and elsewhere, and there is a growing recognition that ‘groups experiencing problems such as homelessness, drug and alcohol misuse, poor mental health, and offending behaviours are often populated to a large extent by the same people’ (Bramley et al., 2020). They often face a ‘tri‐morbidity’ (Cornes et al., 2018); a combination of poor physical health, mental ill health, and problematic substance use (Cornes et al., 2018; Dobson, 2019; Fitzpatrick et al., 2013; Luchenski et al., 2018; Renedo & Jovchelovitch, 2007), and that longer periods of homelessness are associated with greater severity of these issues (Mayock et al., 2011).

It is increasingly recognised that adults experiencing homelessness (particularly those experiencing the more extreme and visible forms of homelessness, such as rough sleeping/being unsheltered) face significant barriers accessing services, and often fall through the cracks between different services they need to access (Dobson, 2019). They often have repeated, but intermittent, contact with a range of publicly funded services, particularly health (Aldridge et al., 2018), criminal justice (Bramley et al., 2020), and local government (Dobson, 2019). For example, this population is five times more likely to attend Accident and Emergency (Emergency Room), and three times more likely to be admitted to hospital, than their housed peers (Cornes et al., 2018). The existing evidence of effectiveness of interventions for this population is mixed (Luchenski et al., 2018), and there is no specific systematic review on the effectiveness of psychosocial interventions. Most of the extant evidence base that examines the effectiveness of interventions around homelessness are focused on individual‐level interventions. They are typically aimed at addressing the harms caused by homelessness or reducing homelessness, rather than prevention.

When targeted at people experiencing homelessness, psychosocial interventions are a group of interventions aimed at addressing the individual‐level causes and consequences of homelessness. These interventions are increasingly used with people experiencing homelessness for a number of reasons. Firstly, there is growing evidence of their effectiveness within the general population, and it is assumed they should therefore be effective for people experiencing homelessness. However, people experiencing homelessness face significant challenges when accessing, maintaining, and benefiting from services compared to the general population, so evidence about the effectiveness of these interventions in general may not be directly translatable to this specific population. The purpose of this review is to systematically identify and synthesise evidence of the effectiveness of psychosocial interventions that is specific to adults experiencing homelessness. Secondly, psychosocial interventions are often used to address clinical needs (such as mental ill health and problematic substance use) which people experiencing homelessness often present. Finally, a number of health bodies (e.g., the National Institute for Health and Care Excellence (NICE) in England and Wales) recommend the use of such interventions.

### 1.2 The intervention

#### 1.2.1 Defining psychosocial interventions

There is a lack of a single, agreed definition of psychosocial interventions (Hodges et al., 2011). In a recent Cochrane systematic review of psychosocial interventions for informal caregivers, Treanor et al. (2019) set out their own definition as ‘focused on non‐pharmacological interventions that were designed to inform, educate and increase the coping capacity’ of the intervention's recipient. In another systematic review about the effectiveness of psychosocial interventions for depression in older people, Forsman et al. (2011) draw on a definition of an earlier systematic review (Ruddy & House, 2005) that ‘any intervention that emphasizes psychological or social factors rather than biological factors’, which they state includes ‘psychological interventions and health education, as well as interventions with a focus on social aspects, such as social support’. Another definition by Jhanjee (2014) states that psychosocial interventions are ‘…a broad array of treatment interventions, which have varied theoretical backgrounds. They are aimed at eliciting changes in the patient's drug use behaviors well as other factors such as cognition and emotion using the interaction between therapist and patient’. The Welsh Government defined psychosocial interventions as: ‘…therapeutic and structured processes, which address the psychological and social aspects of behaviour. The interventions can vary in intensity depending on the needs of individuals’ (Welsh Government, 2011). One broad definition, and the definition that we propose to use for this review, is provided by England et al. (2015) in their report *Psychosocial interventions for mental and substance use disorders: a framework for establishing evidence‐based standards*. They state that psychosocial interventions are ‘interpersonal or informational activities, techniques, or strategies that target biological, behavioral, cognitive, emotional, interpersonal, social, or environmental factors’ which aim to make positive changes to the lives of individuals engaging in these activities.

There are some commonalities underpinning these various definitions. These include interventions have a change objective/aim, and that this intended change is psychological, and is often (though not exclusively) focused on mental ill health or problematic substance use. Several include social change as well as psychological change as an objective, and also all exclude interventions that are wholly or mostly pharmacological in approach. But the extant literature also identifies huge variation in these interventions, including differences in setting, intensity, whether the intervention is group or individual based, and the treatment goals of the intervention. It is also the case that many of the definitions available are broad in scope, so that almost any intervention or service might be considered to be ‘psychosocial’. Undertaking a meta‐analysis that encompasses almost all interventions, covering every form of homelessness, would not be a feasible proposition. It is therefore essential that the review team adds further to the proposed definition of psychosocial interventions, so that we focus on specific interventions. For this review, we propose to use the definition provided by England et al. (2015) and outlined above, and further to focus on psychosocial interventions that are: (a) formally recognised as being psychosocial interventions; (b) are structured or planned, with an explicit intended goal or objective; (c) excludes pharmacological interventions (or interventions that are predominately pharmacological in nature); and (d) targeted for use with adults experiencing homelessness. Given this focus, we have identified a list of 20 interventions that will be the primary focus of this review, which is set out in more detail in Table 1 in the Methods section of this protocol.

#### 1.2.2 Psychosocial interventions and adults experiencing homelessness

Psychosocial interventions are often used to address problematic substance use, poor mental health, and offending behaviours, as well wider social determinants of health such as housing instability and homelessness, worklessness, and poor skills or education. As adults experiencing homelessness will often be dealing with more than one of these issues at any given time, many will access services that use psychosocial interventions. It is therefore essential to understand whether these interventions are effective for adults experiencing homelessness.

#### 1.2.3 How the intervention might work

Broadly speaking, the main mechanism of change underpinning these interventions is psychological, focusing on the individual's psychological development and interaction with their social environment. There is no single theory of change underpinning these types of interventions; some are more explicitly based on formal theories, others less so. England et al. (2015) and others argue that psychosocial interventions draw on different theoretical models. In some areas, there are many different interventions derived from the same theoretical model. They also suggest that a number of interventions are adaptations of other interventions targeting different ages, delivery methods (e.g., individual, group), or settings. At this stage, we draw on the work of Nick Maguire and colleagues (Maguire, 2022) to identify three broad theories of change to understand how psychosocial interventions might work. These theories of change will be further developed and critically evaluated through the early stages of the evidence synthesis. The three broad theories of change are:

- Interpersonal relationships. This assumes that an individual's interactions with other people affect their sense of security, self, motivations, physical health, and behaviours. The idea is that an individual's current relationships drive homelessness, problematic substance use, and mental ill health issues. Psychosocial interventions drawing on this approach focusing on improving interpersonal functioning, providing effective tools for dealing with relationship problems. They also involve providing supportive, non‐judgement support. Family therapy is an example of an intervention that draws on this theory of change.
- Habituation. Habituation assumes that, over time, certain behaviours change from being reward‐driven to be automatized, highly stimulus bound, inflexible, and insensitive to the associated outcomes (positive or negative). Psychosocial interventions drawing on this approach aim to disrupt of change these habits, using approaches such as exposure therapy or contingency rewards.
- Meta cognitive awareness refers to a set of activities which involve thinking about one's thinking and responding accordingly to what is happening in the moment in one's life. Psychosocial interventions drawing on metacognitive awareness approaches focus on cognitive processes and related dysfunctional beliefs or specific cognitive biases. The aim is to help individuals understand how their cognitive biases might lead to, or prolong, homelessness, substance use, and mental health issues, and to provide alternative ways of responding to these thoughts and thereby reduce these symptoms. Motivational interviewing is an example of an intervention that draws on this theory of change.

It is possible that some individual interventions might draw on more than one of these theories of change. As the review team develops and critically assesses these theories of change, it will need to identify which specific interventions draw on which theory, and whether any draw on more than one theory of change.

### 1.3 Why it is important to do this review

#### 1.3.1 Policy relevance

There are ongoing policy and practice debate around the causes of homelessness, and around interventions aimed at preventing and reducing homelessness, or at preventing or reducing issues affecting the health, wellbeing, and social functioning of people experiencing homelessness. Policy makers interested in using the evidence to determine whether and what types of interventions are most effective face considerable challenges in navigating and interpreting the extant effectiveness evidence base. This systematic review aims to provide a single synthesis of the evidence base to aid policy makers in their decisions.

Homelessness is a significant and growing policy issue in a number of countries around the world. It is increasingly recognised that homelessness has a devastating effect on those experiencing it, on the wider community, and is costly to the public purse. There are ongoing debate around which interventions are most effective in preventing and reducing homelessness, particularly in relation to people experiencing severe and multiple disadvantage homelessness.

Psychosocial interventions increasingly play a role in policy responses to homelessness and the harms caused by homelessness. Yet while we do know about the effectiveness of some psychosocial interventions, there is no systematic review that is specific to adults experiencing homelessness. This proposed review will provide policy makers, commissioners, and service providers with insight into the effectiveness of different psychosocial interventions for this specific population.

There is also a significant gap in the extant effectiveness evidence in terms of the voice of people with lived experience of homelessness, and largely treats people with lived experience as passive research participants. This proposed review aims to give voice to people with lived experience in two ways. First, there will be an experts by experience review process that will run alongside the technical peer review process. This will enable the review team to gain views on relevance and appropriateness of the definitions of homelessness and psychosocial interventions underpinning this review, the theories of change identified, and the outcomes used in the underlying studies. We plan to hold several workshops throughout the review to gain feedback from this group. Secondly, the team proposes to work with a panel of people with lived experience to co‐produce the discussion, recommendations, and conclusions of the published review. This panel has been involved in the scoping work in preparing this protocol, through a workshop to discuss definitions of homelessness and psychosocial interventions, the review objectives, and how further to involve individuals with lived experience in the conduct of the review.

#### 1.3.2 Previous reviews

Nick Maguire at the University of Southampton is currently leading a systematic review around the effectiveness of psychological interventions with people experiencing homelessness. The Maguire review is nearing completion, and should be published in 2022. It is not registered with The Campbell Collaboration. Our review team has met with Nick Maguire to discuss the scope and approach of this review. There are two key differences between the Maguire review and the review proposed here. First, the Maguire Review focuses on psychosocial outcomes whereas our proposed review focuses on psychosocial interventions. Secondly, the Maguire Review concentrates on RCT studies, whereas our proposed review also takes account of quasi‐experimental and before and after studies. We are confident that the proposed review will complement that being undertaken by Nick Maguire and his team.

Luchenski et al. (2018) identified the absence of a systematic review on psychosocial interventions specific to the population of people experiencing homelessness. Hunt et al. (2019) published a review on psychosocial interventions for a different but partially overlapping population (individuals with problematic substance use and severe mental ill health), but their review focuses on the effect of these interventions only in relation to substance use. Some specific reviews (considering people experiencing homelessness but on more specific topics such as smoking cessation) have been published recently or are ongoing. A recently published systematic review and meta‐analysis by Hyun et al. (2020) focused on the psychosocial outcomes of psychosocial interventions for adults experiencing homelessness. This review focused on different outcomes to those covered by the proposed review set out here.

## 2 OBJECTIVES

This systematic review is part of a broader evidence synthesis which aims to produce two systematic reviews to address a significant gap in the evidence base identified by Luchenski et al. (2018) and by White (2018). The first review (which is the subject of this protocol) will use meta‐analysis to examine the effectiveness of different psychosocial interventions in (1) reducing problematic substance use; (2) improving mental health; and (3) improving housing stability for adults experiencing homelessness. The second review (which is covered by a separate title registration and protocol) will be of the experiences of adults experiencing homelessness when accessing or using psychosocial interventions, and will be a qualitative evidence synthesis using thematic synthesis (Thomas & Harden, 2008).

This review will have the following questions:

1. How effective are psychosocial interventions in the treatment of adults who are experiencing homelessness?
2. What are the explicit theories of change underpinning psychosocial interventions?
3. Are there differences in the effectiveness of psychosocial interventions in terms of their underlying theories of change?
4. Which type of intervention (e.g., talking therapies, behavioural incentives, self‐help) is most/least effective compared to treatment‐as‐usual?
5. Are there differences in the effectiveness of psychosocial interventions in terms of improving specific outcomes (e.g., housing stability, problematic substance use, mental ill health)?
6. For whom do the interventions work best?

## 3 METHODS

### 3.1 Criteria for considering studies for this review

#### 3.1.1 Types of studies

Eligible studies will be impact evaluations with designs at levels, 3, 4 and 5 of the Maryland Scientific Methods scale,^1^ for example:

- *Level 3. Comparison of outcomes in treated group after an intervention, with outcomes in the treated group before the intervention, and a comparison group used to provide a counterfactual (e.g., difference in difference) … techniques such as regression and (propensity score matching may be used to adjust for difference between treated and untreated groups*
- *Level 4. Quasi‐randomness in treatment is exploited, so that it can be credibly held that treatment and control groups differ only in their exposure to the random allocation of treatment. This often entails the use of an instrument or discontinuity in treatment, the suitability of which should be adequately demonstrated and defended*.
- *Level 5. Reserved for research designs that involve explicit randomisation into treatment and control groups, with Randomised Control Trials (RCTs) providing the definitive example. Extensive evidence provided on comparability of treatment and control groups, showing no significant differences in terms of levels or trends*.

This therefore includes all studies categorised as either ‘Randomised Controlled Trials’ or ‘non‐experimental designs with a comparison group’ from the studies which form the basis of the Homelessness Effectiveness Studies Evidence and Gap Map (EGM) created by the Centre for Homelessness Impact (CHI) and the Campbell Collaboration (Narayanan & White, 2021; Singh & White, 2022a; White, 2018; White et al., 2020).

Studies to be excluded are those with designs at levels 1 and 2 of the Maryland Scientific Methods scale, such as (1) studies without a control or comparison group; (2) ‘before versus after’ designs (without an untreated comparison group); and (3) cross‐sectional regressions.

As the review will therefore essentially include randomised and non‐randomised studies we will undertake a sensitivity analysis to investigate the effect of the inclusion of non‐randomised studies in the meta‐analysis.

### 3.2 Types of participants

There are a number of definitions of homelessness available, reflecting differences between countries and over time. There are also different forms of homelessness, taking into account the length of time someone has been experiencing homelessness, distinctions between living on the street or in their vehicles, or having a temporary place to stay.

We propose to draw on the definition of homelessness used by Keenan et al. (2020) in a recently published Campbell Collaboration protocol. This definition was considered by a workshop of five individuals with lived experienced of homelessness, following which we have slightly adapted and widened the definition. This definition we propose to use for this review is:

Homelessness is defined as those individuals who are in inadequate accommodation (environments which are unhygienic and/or overcrowded), who are sleeping rough (sometimes defined as street homeless), those in temporary accommodation (such as shelters and hostels), those in insecure accommodation (such as those facing eviction or in abusive or unsafe environments), and people whose accommodation is inappropriate (such as those living in tents or vehicles, or ‘sofa surfing’).

Our focus is on adults (men and women aged 18 years and over), undertaken in any high‐income country and published in English. Studies of families or children will be excluded from the review. In many countries (particularly the UK), there are different legal frameworks that apply to families and children experiencing homelessness, and thereby their access to different types of services, and different outcomes expected.

### 3.3 Types of interventions

Given the varying (and often broadly scoped) definitions of what constitutes psychosocial interventions, and the significant differences in whether and how these interventions are structured and delivered, it is important that we be clear about the types of interventions that we will cover in this review.

The review is focused on formal psychosocial interventions used with adults experiencing homelessness, or where at least 40% of the sample is adults experiencing homelessness.^2^ Interventions based solely or mainly on pharmacological approaches will be excluded, as will interventions that might be expected to result in a psychosocial outcome, but are not formally recognised as being psychosocial interventions. The National Institute for Health and Care Excellence (NICE) provides some help here, stating that formal psychosocial interventions include: contingency management, behavioural couples therapy, community reinforcement approach, social behaviour network therapy, cognitive behavioural relapse prevention‐based therapy, and psychodynamic therapy (NICE, 2007).

We draw on this, and have developed a typology of psychosocial interventions to help focus this review. The typology (Table 1) was discussed and validated with an expert panel of academics, policy makers, experts by experience, and practitioners involved in psychosocial interventions targeted at people experiencing homelessness, held in November 2021. This expert panel was convened by the Centre for Homelessness Impact as part of the scoping work undertaken to develop this protocol. The typology will be developed further during the early stages of this systematic review, as individual studies are categorised against the typology. The primary purpose of this, is to categorise studies for eligibility purposes, and to structure the analysis of the effectiveness of individual interventions.

**Table 1:** Proposed typology of psychosocial interventions used with adults experiencing homelessness

Category	Low intensity	High intensity
Talking therapy	Brief interventions	Motivational interviewing
	Brief motivational intervention	Motivational enhancement therapy
	Skills training	Cognitive behavioural therapy
		Dialectical behaviour therapy
		Family therapy/couples therapy/community reinforcement
		Therapeutic communities/residential rehabilitation
		Social behaviour and network therapy
		Psychodynamic therapy
		Relapse prevention
		Mentalisation‐based therapy
	12‐step facilitation therapy	
Behavioural incentives	Contingency management	Community Reinforcement Approach
	Cue exposure treatment	
	Non‐contingent rewards	
Self efficacy /		12 step programmes
Self help/mutual aid		SMART

The typology categorises specific interventions as either low intensity or high intensity, drawing on the distinction made by the Welsh Government (2011) between interventions normally delivered as a single session, and interventions that are formal and structured and delivered over a number of sessions. If feasible, we propose to see whether intensity is an important variable in the effectiveness of psychosocial interventions for adults experiencing homelessness.

The typology further categories interventions by their type. Talking therapies are a type of psychosocial intervention that primarily involves the service user discussing issues around their thoughts, feelings, or behaviours with a professional therapist. These interventions might be delivered in group or one‐to‐one settings.

Behavioural incentives are a type of psychosocial intervention that use extrinsic rewards or negative consequences to change an individual's behaviour. Finally, self‐help interventions are a group of psychosocial interventions in which individuals work through therapeutic materials or processes on their own, or with minimal input from a professional therapist. This can involve working in a group with others also going through the same process. Again, if feasible, we intend to examine whether intervention type is important in terms of the effectiveness of psychosocial interventions (Table 1).

### 3.4 Types of outcome measures

The review will focus on three outcomes associated with psychosocial interventions and which are directly relevant to adults experiencing homelessness. These outcomes are:

1. Housing instability
2. Problematic substance use
3. Mental ill health

As we outline in the background section of this protocol, many people experiencing homelessness face a ‘tri‐morbidity’ of homelessness, substance use, and mental ill health (Cornes et al., 2018). As these are often the three most significant issues facing people experiencing homelessness, it is appropriate to focus this review on whether these interventions generate change in these outcome areas. The review will investigate these outcomes, primarily identifying studies by outcome from 594 studies which are the basis of the Homelessness Effectiveness Studies EGM 4th edition created by the Centre for Homelessness Impact and the Campbell Collaboration (Singh & White, 2022a). We expect these outcomes to be measured in a number of ways by primary studies. These might include:

For problematic substance use:

- Number of days per month substances are used
- Self‐reported measures of drug related problems
- Drug use reduction programme participation
- Drug testing

For housing instability

- Number of days without accommodation
- Number of times without accommodation
- Number of days in stable accommodation
- Number of moves and reason for move

For mental ill health

- Presence and frequency of symptoms of mental ill health
- Severity of mental ill health

To be included, a study must measure changes in outcomes in at least one of these three outcome areas.

As such we expect a range of continuous and binary outcomes to feature in the reviewed studies, and we will convert these into the same metric (e.g., Hedges' g) for meta‐analysis (Borenstein et al., 2009). Where effect sizes are converted from a binary to continuous measure (or vice versa, depending on our ultimate choice of effect size), we will undertake a sensitivity analysis to investigate the effect of the inclusion of studies with a converted effect size in the meta‐analysis.

## 4 SEARCH METHODS FOR IDENTIFICATION OF STUDIES

The primary search method for identification of studies if the Effectiveness EGM 4th edition developed and published by The Campbell Collaboration (Singh & White, 2022a). This EGM includes searches conducted up to September 2021, and was published in April 2022.

The EGM focuses on effectiveness studies, in the form of systematic reviews and impact evaluations. It shows relevant evidence organised into an interactive online matrix capturing where there is evidence for different categories of intervention and how they affect a range of outcomes. The third edition of the effectiveness map includes 394 studies, including 134 new studies identified through an updated search conducted from March to June 2020. The most recent, fourth edition will be published by April 2022 and includes 557 studies, 163 of which were newly identified during an updated search concluding in September 2021. The Effectiveness EGM provides the initial search from which studies for this review will be selected. Specifically, eligible studies will be those with designs at levels, 3, 4 and 5 of the Maryland Scientific Methods scale,^3^ as previously described.

A 5th edition of the Effectiveness EGM, based on searches conducted during the summer of 2022, should become available during the lifetime of this research project. Subject to timing, the review team will identify any studies added to the 5th edition since the 4th edition was published, and review these additional studies for potential inclusion in the systematic review and meta‐analysis.

The process for identifying and searching for the studies included in the EGM list is described by White et al. (2020) in their published *PROTOCOL: Studies of the effectiveness of interventions to improve the welfare of those affected by, and at risk of, homelessness in high income countries: An evidence and gap map*, and for the sake of brevity, we do not repeat it in detail here.

### 4.1 Searching other resources

In January/February 2022 the review team issued a *call for grey evidence* (with a deadline of 28th February 2022) which was disseminated through Manchester Metropolitan University and the Centre for Homelessness Impact social media channels, inviting people with lived experience, researchers, commissioners, service providers and wider stakeholders to submit relevant grey evidence for consideration in the review. Specifically, the call was for evidence that is:

- empirical, based on research that:
  o elevates the voice to people with experience of homelessness;
  o measures the impact of interventions (before and after, quasi‐experimental, randomised controlled trial);
- identifies the barriers to, and facilitators of, successful implementation of interventions;
  o is about psychosocial interventions with outcomes around housing instability, problematic substance use, and mental ill health;
  o is not published in a book or academic journal; and
  o is specific to the UK, or England, Northern Ireland, Scotland or Wales.

The reviewers will also *hand search key journals*, using similar search terms and date ranges as White et al. (2020). While some may have already been searched as part of the EGM (Singh & White, 2022b; White et al., 2020), this targeted journal search and more substance use and treatment focused search will further ensure the capture of all existing literature and evidence. The hand searched journals will include:

- Psychiatric Services Journal
- American Journal of Public Health
- BMJ
- European Journal of Homelessness
- Housing Studies
- Social Policy and Administration
- Journal of Social Distress and Homelessness

## 5 DATA COLLECTION AND ANALYSIS

### 5.1 Description of methods used in primary research

Primary research will be based on designs at levels 3, 4 and 5 of the Maryland Scientific Methods scale, including experimental (randomised) and quasi‐experimental studies. Such studies will measure the effectiveness of an intervention designed to reduce problematic substance use, housing instability, and/or mental ill health against another intervention or a control group (e.g., no intervention, treatment as usual, wait‐list).

### 5.2 Screening

Studies will be selected from the 4th edition of the EGM. Further studies will be added to the shortlist from three sources:

- unpacking systematic reviews contained in the EGM list
- the call for grey evidence
- handsearching key journals

As previously stated, subject to timing and availability, studies added to the Effectiveness EGM in its 5th edition will also be considered for inclusion in this systematic review.

The reviewers will begin to shortlist studies for this review by undertaking title and abstract screening of each individual study: (a) listed in the Effectiveness EGM; (b) identified through the call for evidence; and (c) identified through hand searches. This title and abstract screening will be undertaken independently by three reviewers, and any disagreements will be escalated for adjudication to a subject matter expert or methods expert on the review team.

Systematic reviews from the EGM list that are identified as being relevant at title and abstract stage will be unpacked. This list of studies will be checked against the Effectiveness EGM, and any duplicates will be removed. The remaining studies (i.e., studies included in relevant systematic reviews that do not appear in the Effectiveness EGM) will also be subjected to a title and abstract search against the review's inclusion and exclusion criteria. Again, the title and abstract screening of will be conducted by three reviewers independently, and any disagreements will be escalated for adjudication to a subject matter expert or methods expert on the review team.

Once the title and abstract screening is complete, each potentially includable study will then be full text screened using the inclusion/exclusion criteria previously defined. All screening will be undertaken by two reviewers, and any disagreements will be escalated to a subject matter and a methods expert on the review team. Twenty‐five percent (25%) of final screening decisions will be sampled by a third reviewer. Final decisions about inclusion will be made by all members of the review team.

Following the two levels of screening, a list of studies eligible for inclusion in this systematic review will be agreed before data extraction for meta‐analysis is undertaken.

### 5.3 Data extraction and management

Data will be extracted from eligible studies by two reviewers, to include details of the study, quantitative data required for meta‐analysis, and confidence in the study's findings (using the Campbell Collaboration's critical appraisal tool for primary studies—White et al., 2020, p. 11). Coding disagreements will be discussed and if necessary passed to the lead reviewer for resolution. We will extract data for the following, where it is not already present in the Effectiveness EGM:

- Publication details (e.g., authors, year, source, study location)
- Intervention details
- Theory of change classification
- Intervention classification
- Participant details, including classification (e.g., age, gender)
- Study design
- Comparison (e.g., other intervention, treatment as usual, waitlist control)
- Outcome description, definition and measurement (including measurement duration)
- Sample sizes of treatment and control groups
- Data to calculate Odds Ratios or Standardised Mean Difference (SMD)
- Confidence assessment

### 5.4 Assessment for confidence in the included studies

Studies selected for this review that are included in the EGM have been assessed for confidence of findings using two separate critical appraisal tools: one for primary studies and one for systematic reviews. Details of these tools and the assessment process are set out in White et al. (2020). The critical appraisal tool used for primary studies is one developed by the Campbell Collaboration for use with maps and reviews. The tool for primary studies has seven items which relate to (1) study design, (2) blinding, (3) power calculations, (4) attrition, (5) description of the intervention, (6) outcome definition and (7) baseline balance. Each of these seven items is rated as implying high, medium or low confidence in study findings. Overall quality is assessed using the ‘weakest link in the chain’ principle, so that confidence in study findings can only be as high as the lowest rating given to any of the critical items.

Systematic reviews included in the Effectiveness EGM by the Campbell team were assessed using AMSTAR 2 (‘Assessing the Methodological Quality of Systematic Reviews’). This checklist has 16 items which cover: (1) PICO in inclusion criteria, (2) ex ante protocol, (3) rationale for included study designs, (4) comprehensive literature search, (5) duplicate screening, (6) duplicate data extraction, (7) list of excluded studies with justification, (8) adequate description of included studies, (9) adequate risk of bias assessment, (10) report sources of funding, (11) appropriate use of meta‐analysis, (12) risk of bias assessment for meta‐analysis, (13) allowance for risk of bias in discussing findings, (14) analysis of heterogeneity, (15) analysis of publication bias, and (16) report conflicts of interest. As previously stated, any relevant systematic review from the Effectiveness EGM will be unpacked and the any primary studies included will be assessed for inclusion in our review.

The assessments undertaken by the Campbell Collaboration form the basis of our quality assessment and we do not propose to undertake further assessment on these studies. Studies that identified through the additional searches outlined above, or any studies unpacked from systematic reviews, which are not listed in the EGM and therefore have not been assessed will be subjected to assessment of confidence, using this tool. Table 2 summarises our proposed approach to assessment for confidence in included studies.

**Table 2:** How studies will be assessed for confidence in findings

Source of included study	How study is assessed for confidence
Primary studies from Effectiveness EGM	Assessed by Campbell team as part of EGM work and will not be assessed by our review team
Primary studies unpacked from systematic reviews in the Effectiveness EGM	Primary studies that are in the EGM have been assessed by Campbell team as part of EGM work and will not be assessed by our review team
	Primary studies that are not in the EGM will be assessed by our review team using Campbell's Critical Appraisal Tool
Primary studies identified through hand searches or the call for evidence	Primary studies that are in the EGM have been assessed by Campbell team as part of EGM work and will not be assessed by our review team
	Primary studies that are not in the EGM will be assessed by our review team using Campbell's Critical Appraisal Tool

Where studies are assessed for confidence by our review team, classifications will be undertaken by one researcher and judgements (high/medium/low confidence) will be verified by a second researcher who will sample check 25%. Sensitivity analyses will be undertaken to determine the effect of excluding studies classified as low confidence, effectively by running an additional analysis with these studies omitted (see section below).

### 5.5 Criteria for determination of independent findings

Dependent effects can occur when a study reports results for multiple measures of the same outcome construct for the same sample, the same outcome measure at multiple time points, when a study has multiple treatment arms compared to a common control group, or multiple studies evaluate the same programme and report on the same outcome. This is problematic as estimating an average effect using standard meta‐analytic models rely on the statistical assumption of independence of each included effect size (Gleser & Olkin, 2007).

Once we have identified our pool of included studies, we will map the programmes/interventions being evaluated, the outcome measures used in each study and the follow‐up time(s) to identify possible dependent effects. We will implement the following strategies to address dependent effects, drawing on (Pigott & Polanin, 2020), with an aim to balance capturing as much relevant information from each study as possible with the limited timeframe for the review:

- Multiple follow‐up points: If a study reports results for the same outcome at multiple follow‐up points, we will extract data for a maximum of two time points and calculate standardised effect sizes for both.
- Multiple measures of the same construct: if a study reports results for multiple outcomes measuring the same or similar construct, we will extract one measure within two groups of outcome measures from each study, using the most commonly used measures across the selected studies. Within these two categories of outcomes, if there are multiple self‐report measures or multiple objective measures in a single study, we will decide on which to extract based on which is most common across the review.
- Multiple studies on the same programme: if different studies report on the same programme but use different samples (e.g., from different regions), we will include all in the review and include both in the same meta‐analysis, treating them as independent samples. This is provided that the effect sizes were measured relative to a different control group. If multiple studies report on the same outcome(s) with overlapping samples, we will choose the study with the larger sample size for inclusion in the review.
- Multiple treatment arms compared to a common comparison group: if a study reports results for multiple treatment arms and all interventions meet our inclusion criteria, we will extract data for all arms and include in the same meta‐analysis using robust variance estimation (as described above).
- Multiple specifications: if a study reports multiple estimates using different specifications for the same outcome, we will choose the one that the authors present as their primary estimate. We will prioritise the Intention To Treat (ITT) estimate from RCTs where possible as a more conservative and realistic estimate of programme impact.
- Multiple papers on the same study: if we identify multiple reports on the same study (e.g., a journal article and a working paper), we will include the most recent version.

### 5.6 Measures of treatment effect

Effect sizes will be calculated using the esc package in R (effect size computation for meta analysis) (Ludecke, 2019) and Wilson's Practical Meta‐Analysis Effect Size Calculator (Wilson, 2017). If a study does not provide sufficient raw data for the calculation of an effect size we will attempt to contact the author(s) to obtain this data. Where effect sizes are converted from a binary to continuous measure (or vice versa), we will undertake a sensitivity analysis to investigate the effect of the inclusion of studies with a converted effect size in the meta‐analysis by running an additional analysis with these studies omitted (see section on sensitivity analysis). If a study includes multiple effects we will carry out a critical assessment to determine (even if only theoretically) whether the effects are likely to be dependent. Where we suspect dependent effects we will determine whether we can account for these by the Correlated and Hierarchical Effects (CHE) model (Pustejovsky & Tipton, 2022) in combination with Robust Variance Estimation, using the metafor (Viechtbauer, 2010) and clubSandwich (Pustejovsky & Tipton, 2022) packages in R. This model assumes that there is a constant correlation among groups of effect sizes from the same study, and also allows for between‐study and within‐study heterogeneity in true effect sizes. We will also as far as possible extract consistent measurements across. We will also assess for unit of analysis errors, and based on the number of studies which feature such errors we will make a decision about whether to exclude these studies from any meta‐analysis.

### 5.7 Data synthesis

It is likely that study interventions and comparison groups will be heterogeneous and complex. We will first determine whether meta‐analysis is possible by making a judgement about the similarity of interventions to be synthesised. If they are too heterogeneous for inclusion in meta‐analysis we will undertake a narrative synthesis.

Should meta‐analysis be feasible, all analyses will be undertaken using the statistical programming language R, principally using the metafor package (Viechtbauer, 2010), and the clubSandwich package for use of the cluster‐robust variance estimators with small‐sample corrections (Pustejovsky & Tipton, 2022). Random effects models and restricted maximum‐likelihood estimation will be used to estimate the total amount of heterogeneity. Random effects models are appropriate when the constituent studies differ in terms of mixes of participants and interventions (Borenstein et al., 2009). All analyses will include an assessment of statistical heterogeneity, that is, variability in intervention effects. In meta‐analyses of social interventions, we expect statistical heterogeneity to be substantial and driven by variability in the underlying studies, which are likely to represent a range of interventions delivered to participant groups with different characteristics, in different locations and at different times (Table 3).

**Table 3:** Proposed analyses by research objective

Research question	Analytic approach
How effective are psychosocial interventions in the treatment of adults who are experiencing homelessness?	Meta‐analysis with robust variance estimation, of all studies included in the review.
Are there differences in the effectiveness of psychosocial interventions in terms of their underlying theories of change?	Meta‐analysis with robust variance estimation of all studies included in each intervention group. A subset of studies which can be clearly defined on their theory of change will be used. We therefore to expect to exclude some studies from this analysis, these being of interventions which do not have an explicit theory of change, or which draw on more than one theory of change.
What is the effect of intervention types (e.g., talking therapies, behavioural incentives, self‐help) in terms of improving specific outcomes (e.g., reducing occurrence and/or severity of housing instability, problematic substance use, mental ill health) compared to treatment as usual?	Robust variance estimation meta‐analysis of all studies in each category. If there are insufficient studies to undertake meta‐analysis, we will undertake a narrative synthesis within each intervention category.
What is the effect of individual interventions in terms of improving specific outcomes compared to treatment as usual?	Robust variance estimation meta‐analysis of all studies in each category. If there are insufficient studies to undertake meta‐analysis, we will undertake a narrative synthesis within each intervention category.
Are there differences in the effectiveness of intervention types in terms of improving specific outcomes?	Pairwise comparison of intervention types using robust variance estimation meta‐analysis of all studies in each outcome category. If there are insufficient studies to undertake meta‐analysis, we will undertake a narrative synthesis within each intervention category.
For whom do the interventions work best?	Meta‐regression using robust variance estimation meta‐analysis including all studies with group characteristic as a moderator (gender, race) or sub‐group analysis.

### 5.8 Dealing with missing data

Missing data will be sought as described above, that is, by attempting to contact study author(s) to obtain it. If we are unable to do this then the study will potentially be excluded from analyses, depending on the type of data which is missing.

### 5.9 Assessment of heterogeneity

We will report the effect size and confidence interval for each individual study, and the total amount of heterogeneity (*I* ^2^) for each analysis. This will describe ‘the percentage of variation across studies that is due to heterogeneity rather than chance’.^4^ We will also present tau‐squared (*τ* ^2^) for each meta‐analysis, the estimate for the true variance in effect size among the set of included studies. Since the analysis is using a random effects model, we will also report the prediction interval which allows a more accessible interpretation of the potential range of effect sizes for each intervention (Borenstein et al., 2017).

### 5.10 Assessment of publication biases

As our search strategy includes grey literature, this should help to mitigate any publication bias which might be observed if we were to only include published studies (as published studies are likely to report larger than average effects [Borenstein et al., 2009]). We will however undertake additional analysis to assess whether publication bias is likely to be a factor in our findings. This will include a funnel plot to determine whether the summary effects of the meta‐analysis are subject to publication bias, and if this appears to be the case, further tests (e.g., Duval and Tweedie' Trim and Fill) to determine a ‘best estimate of the unbiased effect size’ (Borenstein et al., 2009). We will also control for effect size dependency by implementing Egger's regression test using robust variance estimation (Pustejovsky & Rodgers, 2019).

### 5.11 Moderator analysis and investigation of heterogeneity

A key goal of meta‐analysis is to identify and analyse heterogeneity in studies included in a review, by exploring whether characteristics of the included studies are associated with variation in effect size. We will therefore carry out moderator analysis around each main outcome cluster (e.g., reducing occurrence and/or severity of housing instability, problematic substance use, mental ill health), primarily through meta‐regression, to explore observed heterogeneity between the included studies if there are sufficient effect sizes and studies to do so. These analyses will be based on the following categorical moderating variables, assuming sufficient data or the required quality can be extracted:

- Intervention type (e.g., talking therapies, behavioural incentives, self‐help)
- Intensity of intervention (low, high)
- Demographics (e.g., gender, race)
- Study location (USA, non‐USA)
- Measurement duration
- Study design (randomised, non‐randomised)
- Confidence in study findings (low, medium, high)

Depending on the number of effect sizes and number of included studies and variation across studies on the characteristics of interest, we will aim to include these moderating variables in a single model. Analyses will be based on random effects, and will be undertaken using the metafor package in R (Viechtbauer, 2010). Again, dependent effects will be accounted for by the Correlated and Hierarchical Effects (CHE) model (Pustejovsky & Tipton, 2022) in combination with Robust Variance Estimation. If there are insufficient number of effect sizes included in the review to undertake meta‐regression, we will attempt single characteristics sub‐group analysis. Any conclusions drawn from the meta‐regression analysis will be cautious and exploratory given that these relationships are observational in nature and likely based on a small number of effects.

### 5.12 Sensitivity analysis

We will undertake sensitivity analysis to determine the effect on our overall findings of:

- Non‐randomised studies
- Studies with effect sizes which have been converted from binary to continuous (or vice versa)
- Studies classified as low confidence in findings

Sensitivity analyses will be undertaken by repeating the meta‐analysis, omitting in turn each of the groups of studies described above, to determine their effect on overall findings.

## CONTRIBUTIONS OF AUTHORS

Review lead: Chris O'Leary

Content: Chris O'Leary, Ligia Teixeira

Systematic review methods: Esther Coren

Statistical analysis: Esther Coren, Zsolt Kiss

Information retrieval: Anton Roberts and Harry Amitage

## DECLARATIONS OF INTEREST

No conflicts of interest.

## PRELIMINARY TIMEFRAME

Review to be completed by March 2023.

## PLANS FOR UPDATING THIS REVIEW

Dependant on additional funding.

## Supporting information

Supporting information.

## Footnotes

1 Level descriptions taken from https://whatworksgrowth.org/resources/the-scientific-maryland-scale/.

2 This criteria was used in the Maguire Review and is considered an appropriate threshold to ensure that study findings are relevant to our population of interest.

3 Level descriptions taken from https://whatworksgrowth.org/resources/the-scientific-maryland-scale/.

4
https://www.statsdirect.com/help/meta_analysis/heterogeneity.htm.
