# Supplementary material for: PROTOCOL: The effectiveness of psychosocial interventions for reducing problematic substance use, improving mental health, and improving housing stability for adults experiencing homelessness: A systematic review and meta‐analysis
Source: Campbell Syst Rev. 2022 Nov 25;18(4):e1290. doi: 10.1002/cl2.1290 (PMC9695752; doi:10.1002/cl2.1290)
Supplement: Supplementary file 1 — Supporting information. [file CL2-18-e1290-s001.docx]

**Appendix A. Search details from White et al., (2019): PROTOCOL: Studies of the effectiveness of interventions to improve the welfare of those affected by, and at risk of, homelessness in high‐income countries: An evidence and gap map**

**Databases**:

1. Academic databases

• Econlit

• The National Bureau of Economic Research (NBER)

• Social Science Research Network (SSRN)

• International Bibliography of Social Sciences (IBSS)

• Applied Social Sciences Index and Abstracts (ASSIA)

• Social Service Abstract

• Embase

• PubMed

• PsycINFO

• MEDLINE

• WHO’s Global Health Library

• CABI’s Global Health

• ERIC

• CINHAL

• SCOPUS

• Web of Science

• EPPI Centre Evaluation Database of Education Research

2. Evidence and Gap Map Database

• 3ie Evidence and Gap Map Repository

• Global Evidence Mapping Initiative

• Evidence‐Based Synthesis Program (Department of Veteran

Affairs)

3. Systematic review databases

• Swedish Agency for Health Technology Assessment and

Assessment of Social Services

• Collaboration for Environmental Evidence

• Cochrane

• Cochrane

• Campbell

• 3ie Systematic Review Database

• Research for Development

• Epistemonikos

4. Trials registries

AEA Social Science RCT Registry https://www.socialscience

registry.org/.

**Grey literature and websites:**

Homeless Hub https://www.homelesshub.ca/

European observatory on homelessness https://www.feantsaresearch.org/en/publications

United State interagency council on homelessness http://www.usich.gov/

EThOS http://ethos.bl.uk/Home.do

WHO ICTRP http://apps.who.int/trialsearch/

Focus on Prevention http://www.preventionfocus.net/

Social Policy and Practice http://www.spandp.net/

10,000 home campaigns https://en.wikipedia.org/wiki/100,000_Homes_Campaign

Anti‐poverty committee https://en.wikipedia.org/wiki/Anti‐Poverty_Committee

Back on my feet https://en.wikipedia.org/wiki/Back_on_My_Feet_(non‐profit_organization)

Feantsa https://www.feantsa.org/

National Coalition Homeless https://nationalhomeless.org/

Homelessness Australia https://www.homelessnessaustralia.org.au/

Mission Australia https://www.missionaustralia.com.au/publications/position‐statements/homelessness

National Alliance to end homelessness https://endhomelessness.org/

Institute of global homelessness https://www.ighomelessness.org/

Homelessness link https://www.homeless.org.uk/

Crisis https://www.crisis.org.uk/about‐us/how‐we‐work/

Housing first https://housingfirsteurope.eu/about‐the‐hub/

Canadian Alliance to end homelessness https://housingfirsteurope.eu/about‐the‐hub/

Social work and policy institutes http://www.socialworkpolicy.org/research/homelessness.html

Association of housing advice services https://www.ahas.org.uk/

Centre point <https://centrepoint.org.uk/>

Homelessness trust funds https://housingtrustfundproject.org/htf‐elements/homeless‐trust‐funds/

Meliville charitable trust https://melvilletrust.org/category/resourcesreports/

Conrad H Hilton foundation https://www.hiltonfoundation.org/priorities/homelessness#resources

Abt Associates https://www.abtassociates.com/

Mathematica https://www.mathematica‐mpr.com/

American Institutes of Research https://www.air.org/

Rand https://www.rand.org/

MDRC https://www.mdrc.org/

**Additional searches using Google and Google Scholar.**

**SAMPLE SEARCH STRING**

Search string/keywords (for ovid medline platform)

**Study design key words**

“quasi experiment*” or quasi‐experiment* or “random* control*

trial*” or “random* trial*” or RCT or (random* adj3 allocat*) or

matching or “propensity score” or PSM or “regression discontinuity”

or “discontinuous design” or RDD or “difference in difference*” or

difference‐in‐difference* or “diff in diff” or “case control” or cohort or

“propensity weighted” or propensity‐weighted or “interrupted time

series” or (before adj5 after) or (pre adj5 post) or ((pretest or pre

test) and (posttest or post test)) or “research synthesis” or “scoping

review” or “rapid evidence assessment” or “systematic literature

review” or “Systematic review” or “Meta‐analy*” or Metaanaly* or

“meta analy*” or “Control* evaluation” or “Control treatment” or

“instrumental variable*” or heckman or IV or (quantitative or

“comparison group*” or counterfactual or “counter factual” or

counter‐factual or experiment*) adj3 (design or study or analysis))

or QED or evaluation).ti,ab,kw

– OR

– clinical trial/or clinical trial, phase i/or clinical trial, phase ii/or clinical

trial, phase iii/or clinical trial, phase iv/or controlled clinical trial/or

randomised controlled trial/or pragmatic clinical trial/

– controlled clinical trials as topic/or non‐randomised controlled

trials as topic/or randomised controlled trials as topic/or

pragmatic clinical trials as topic/or case‐control studies/or

retrospective studies/or controlled before‐after studies/or interrupted

time series analysis/or random allocation/or cohort

studies/or follow‐up studies/or longitudinal studies/or prospective

studies/or retrospective studies/or propensity score/or regression

analysis/or evaluation studies/or matched‐pair analysis

– (“quasi experiment*” or quasi‐experiment* or “random* control* trial*”

or “random* trial*” or RCT or (random* adj3 allocat*) or matching or

“propensity score” or PSM or “regression discontinuity” or “discontinuous

design” or RDD or “difference in difference*” or difference‐indifference*

or “diff in diff” or “case control” or cohort or “propensity

weighted” or propensity‐weighted or “interrupted time series” or

(before adj5 after) or (pre adj5 post) or ((pretest or pre test) and

(posttest or post test)) or “research synthesis” or “scoping review” or

“rapid evidence assessment” or “systematic literature review” or

“Systematic review” or “Meta‐analy*” or Metaanaly* or “meta analy*”

or “Control* evaluation” or “Control treatment” or “instrumental

variable*” or heckman or IV or ((quantitative or “comparison group*”

or counterfactual or “counter factual” or counter‐factual or experiment*)

adj3 (design or study or analysis)) or QED).ti,ab,kw.

(“meta regression” or “meta synth*” or “meta‐synth*” or “meta analy*”

or “metaanaly*” or “meta‐analy*” or “metanaly*” or “metaregression” or

“metaregression” or “methodologic* overview” or “pool* analys*” or “pool*

data” or “quantitative* overview” or “research integration”).ti,ab,sh.

OR

(review adj3 (effectiveness or effects or systemat* or synth* or

integrat* or map* or methodologic* or quantitative or evidence or

literature)).ti,ab,sh.

**Homelessness keywords**

– homeless persons/or homeless youth

– (evict* or homeless* or "housing excl*" or “residential stability”

or ((street* or private or improvised or shelter* or emergency or

temporary or insecure or overcrowded or precarious or stable or

marginal*) adj3 (dwell* or house* or housing or accommodation))

or (street adj3 (life or living or lives or youth* or child* or people or

person*)) or runaway* or “Run away from home” or “Running away”

or “Ran away” or “Going missing” or “Bag lady” or Houseless* or

Unhoused or “without a roof” or Roofless or (rough adj3 sleep*) or

Destitut* or “Skid row*” or “sleepers out”).ti,ab,kw.

– (“Housing first” or “Pathways to Housing” or “Homeless Veterans

Reintegration Program” or “Access to Community Care and Effective

Services and Supports” or 'Support* Housing Program” or “Housing

and Urban Development–Veterans Affairs Supported Housing program”

or “HUD‐VASH” or “Sober Transitional Housing and Employment

Project” or “sober house placement*” or “Housing ladders” or

“Staircase housing” or “low threshold housing” or “Critical Time

Intervention”).ti,ab,kw
